# HOXB9 enhances the ability of lung cancer cells to penetrate the blood-brain barrier

**DOI:** 10.18632/aging.202324

**Published:** 2020-12-19

**Authors:** HongShan Zheng, ChenLong Li, ZhenZhe Li, KaiBin Zhu, HongBo Bao, JinSheng Xiong, Peng Liang

**Affiliations:** 1 Department of Neurosurgery, Harbin Medical University Cancer Hospital, Harbin 150001, Heilongjiang, P.R. China

**Keywords:** brain metastasis, non-small cell lung cancer, blood-brain barrier, HOXB9, epithelial-to-mesenchymal transition

## Abstract

Even after multimodal therapy, the prognosis is dismal for patients with brain metastases from non-small cell lung cancer (NSCLC). Although the blood-brain barrier (BBB) limits tumor cell penetration into the brain parenchyma, some nevertheless colonize brain tissue through mechanisms that are not fully clear. Here we show that homeobox B9 (HOXB9), which is commonly overexpressed in NSCLC, promotes epithelial-to-mesenchymal transition (EMT) and tumor migration and invasion. Animal experiments showed that HOXB9 expression correlates positively with the brain metastatic potential of human NSCLC cells, while brain metastatic cells derived through *in vivo* selection showed greater HOXB9 expression than their cells of origin. Comparable results were obtained after immunohistochemical analysis of clinical primary NSCLC and matched brain metastasis samples obtained after surgery. Using an *in vitro* BBB model, knockdown and overexpression experiments showed that HOXB9-dependent expression of MMP9 in NSCLC cells leads to reduced expression of junctional proteins in cultured human vascular endothelial cells and enhanced transmigration of tumor cells. These data indicate that HOXB9 enables NSCLC cells to break away from the primary tumor by inducing EMT, and promotes brain metastasis by driving MMP9 production and degradation of intercellular adhesion proteins in endothelial cells comprising the BBB.

## INTRODUCTION

It is estimated that 20% of patients with cancer will develop brain metastases, the most common intracranial tumors in adults [1, 2]. Although virtually all cancers can metastasize to the brain, the most common primary tumors associated with brain metastases are lung cancer (20–56% of patients), breast cancer (5–20% of patients) and melanoma (7–16% of patients) [3–5]. Non-small cell lung cancer (NSCLC) is the most common form of lung cancer. More than 50% of patients with advanced NSCLC suffer from brain metastases during disease progression; in these cases, even after multimodal treatment prognosis remains very poor [6–8]. Brain metastases from lung cancer are commonly detected shortly after diagnosis of the primary tumor (4.5 months on average) and often develop synchronously with it [9]. If left untreated, newly diagnosed brain metastasis from NSCLC carries a mean survival time of no more than 2 months [10], which may be extended for only a few months on average by palliative therapies such as corticosteroids, chemotherapy, and radiotherapy [11].

The formation of brain metastases depends on the ability of circulating tumor cells to successfully penetrate the blood brain barrier (BBB) [12]. Many genes and signal transduction pathways have been associated with the spread of lung cancer cells to distant organs, including the brain [13–17]. EGFR mutations and ALK gene rearrangements are particularly important because of the high brain transmission rate conferred by these entities [18, 19]. In addition, inactivation of the tumor suppressor gene LKB1 and activation of the KRAS oncogene greatly influence both lung cancer formation and growth and the risk of developing brain metastases [20]. Still, since the specific mechanisms remain unclear, identifying genes and pathways related to BBB breakdown and infiltration of tumor cells into the brain is essential to develop new therapeutic strategies to prevent brain metastasis in lung cancer patients.

HOXB9 is a member of the homeobox gene (HOX) family of transcriptional regulators that act as key controllers of cell proliferation and differentiation during embryonic development [21]. Accordingly, HOXB9 has been shown to regulate cell growth, tissue remodeling, and stem cell self-renewal [22], and its expression has been also associated with tumor development and chemoresistance. However, both oncogenic and tumor suppressor functions have been attributed to HOXB9 in different cancers [23–27]. For example, HOXB9 was shown to act as an oncogene in breast cancer by inducing the expression of angiogenic factors and promoting lung metastasis [28]. In contrast, HOXB9 might play a tumor suppressor function in gastric adenocarcinoma cells, as its overexpression increases apoptosis and inhibits metastasis formation [27]. High expression of HOXB9 has been associated with poor prognosis in NSCLC [29]. A study has shown that signaling through the WNT/TCF pathway in lung cancer promotes metastatic dissemination to brain and bone by downstream activation of HOXB9 and LEF1 genes [30]. Meanwhile, another study showed that PCAF-mediated acetylation of HOXB9 inhibited lung cancer progression [31]. Considering the critical role of HOXB9 in lung cancer progression and metastasis, we examined its expression in clinical NSCLC samples and cell lines and conducted knockdown and overexpression experiments to evaluate its impact on BBB breakdown and brain metastasis formation.

## RESULTS

### HOXB9 is overexpressed in NSCLC cells

**Figure 1:**
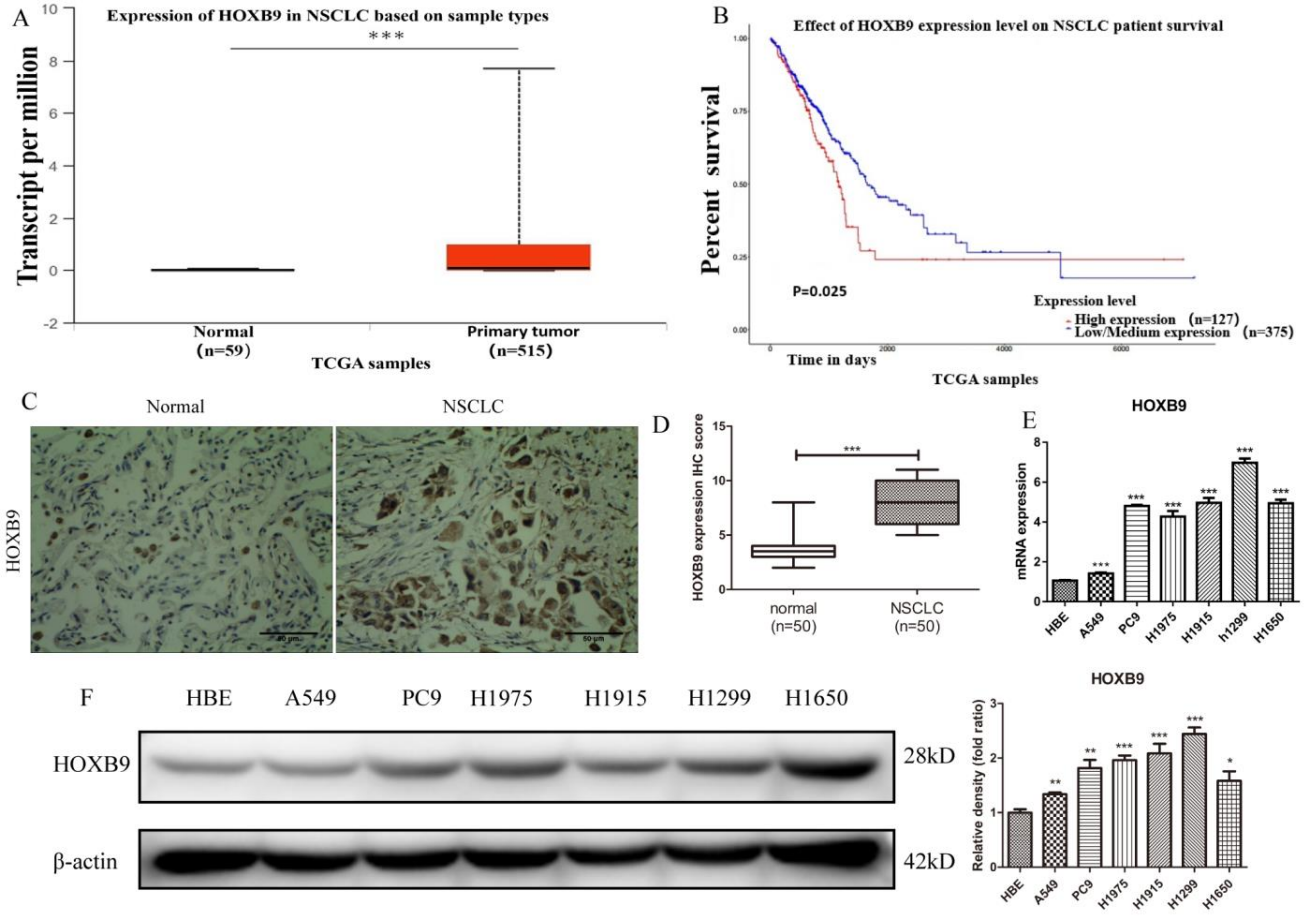
**HOXB9 is highly expressed in clinical NSCLC specimens and NSCLC cell lines and correlates with shorter patient survival.** (**A**) Analysis of HOXB9 expression in NSCLC (n = 515) and normal lung tissue samples (n = 59) retrieved from the TCGA database. HOXB9 expression was significantly higher in NSCLC specimens (p<0.001). (**B**) High-HOXB9 expression is correlated with shorter overall survival (OS) in NSCLC (TCGA data). High-HOXB9 expression group, n = 127; low-HOXB9 expression group, n = 375; P = 0.025. (**C**) Representative images of HOXB9 expression from IHC analysis of 50 matched NSCLC and normal lung samples. Scale bars = 50 μm. (**D**) IHC score-based quantification of total HOXB9 IHC data from (**C**); HOXB9 expression was significantly higher in NSCLC samples (p<0.001). (**E**) Relative HOXB9 mRNA expression in NSCLC cell lines and human bronchial epithelial cells (HBE). (**F**) Western blotting analysis of HOXB9 expression in NSCLC cell lines and HBE cells, and the gray level analysis results for western blotting; *p < 0.05, **p < 0.01, ***p < 0.001.

Analysis of clinical NSCLC samples and normal lung tissues in the TCGA database showed that HOXB9 was highly expressed in NSCLC (Figure 1A), and correlated with lower overall survival (OS) (Figure 1B). HOXB9 expression results were validated by IHC in matched NSCLC and normal lung samples (n = 50) from patients with NSCL (Figure 1C, 1D). Using a human bronchial epithelial cell line (HBE) as control, we also confirmed by western blot and qPCR high expression of HOXB9 in several NSCLC cell lines (Figure 1E, 1F). These findings would suggest that HOXB9 has tumor-promoting functions and may confer poor prognosis in NSCLC.

### HOXB9 promotes migration and invasion in NSCLC cells

**Figure 2:**
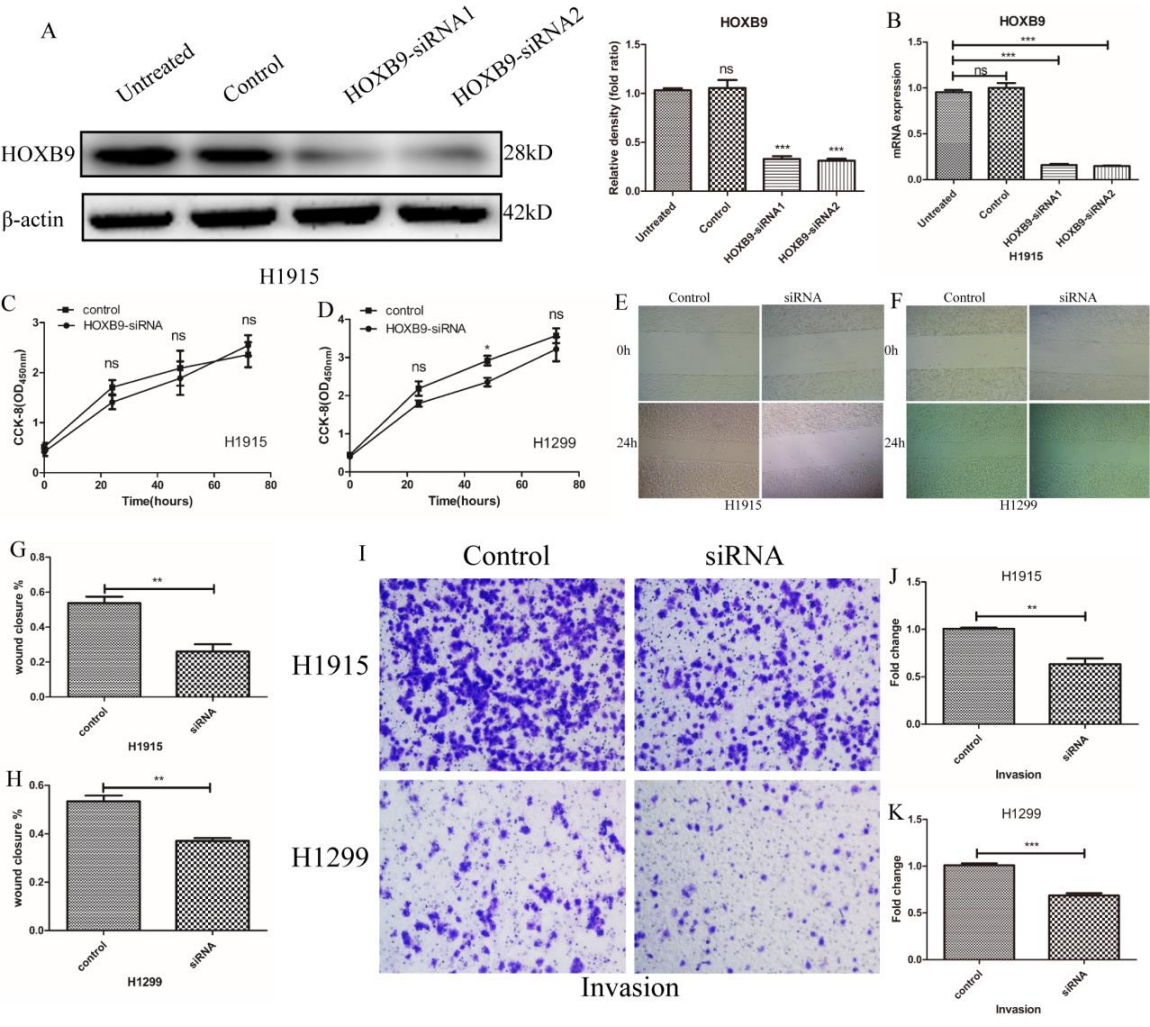
**HOXB9 promotes migration and invasion in NSCLC cells.** (**A**, **B**) HOXB9 knockdown was performed in H1915 and H1299 NSCLC cells by transfection of HOXB9-siRNA. Scrambled siRNA was used as negative control. HOXB9 knockdown efficiency was determined using western blot, gray level analysis and qPCR. (**C**, **D**) Cell proliferation was determined by the CCK-8 assay in H1915 and H1299 cells at 24, 48, and 72 h after transfection with HOXB9-siRNA or scrambled siRNA. HOXB9 knockdown did not affect proliferation in any cell line. (**E**, **F**) Representative images from wound-healing migration assays in H1915 and H1299 cells. (**G**, **H**) Quantification of wound-healing assay results shows decreased migratory potential after siRNA-mediated HOXB9 knockdown (p = 0.0036 and p = 0.0079 for H1915 and H1299 cells, respectively). (**I**) Representative images from Transwell invasion assays in H1915 cells and H1299 cells. (**J**, **K**) Quantification of Transwell invasion assay data showing that HOXB9 knockdown significantly inhibited invasive potential in both cell lines (p = 0.0037 and p = 0.0005 for H1915 and H1299 cells, respectively).

Knockdown experiments were conducted in two NSCLC cell lines showing high HOXB9 expression (H1915 and H1299) by transfecting HOXB9-siRNA (Figure 2A, 2B). CCK-8 proliferation assays showed no significant growth differences between HOXB9-knockdown cells and control cells transfected with scrambled siRNA up to 72 h post-transfection (Figure 2C, 2D). In contrast, both migration and invasion, assessed respectively by wound healing (Figure 2E–2H) and Transwell Matrigel (Figure 2I–2K) assays, were markedly decreased 24 h after HOXB9 knockdown.

**Figure 3:**
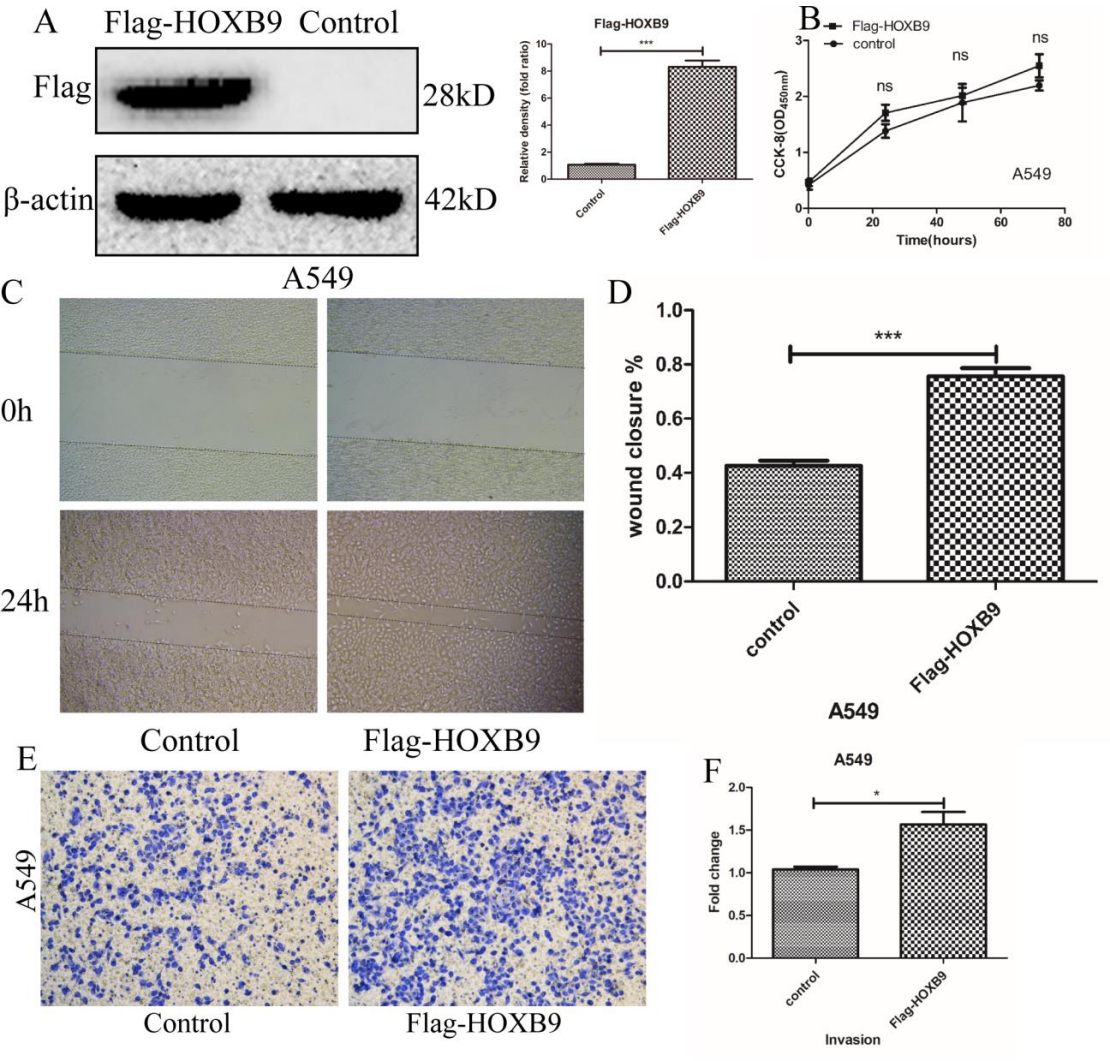
**HOXB9 overexpression promotes migration and invasion, but not proliferation, in A549 cells.** (**A**) A vector encoding Flag-HOXB9 was used to induce HOXB9 overexpression in A549 cells. An empty vector served as control. HOXB9 overexpression was confirmed by western blot and gray level analysis. (**B**) CCK-8 assay results showing no significant differences in cell proliferation between Flag-HOXB9 and control A549 cells. (**C**) Wound-healing closure images of Flag-HOXB9 and control A549 cells. (**D**) Quantification of results from wound-healing assays shown in (**C**); p = 0.0007. (**E**) Transwell assays comparing invasion potential between control and HOXB9-overexpressing A549 cells. (**F**) Quantification of results from experiments shown in (**E**); p = 0.0238.

Next, we overexpressed HOXB9 in A549 cells (showing low HOXB9 expression) by transfecting vectors encoding Flag-HOXB9 (Figure 3A). Consistent with the above results, experiments showed that HOXB9 overexpression did not affect cell proliferation (Figure 3B), whereas migration and invasion were both increased significantly (Figure 3C–3F). In addition, we performed colony-forming assays which confirmed that HOXB9 expression does not influence proliferation in NSCLC cells (Supplementary Figure 1A, 1B). Preliminary results indicate that HOXB9 promotes the migration and invasion of non-small cell lung cancer.

### HOXB9 promotes brain metastasis formation by NSCLC cells

**Figure 4:**
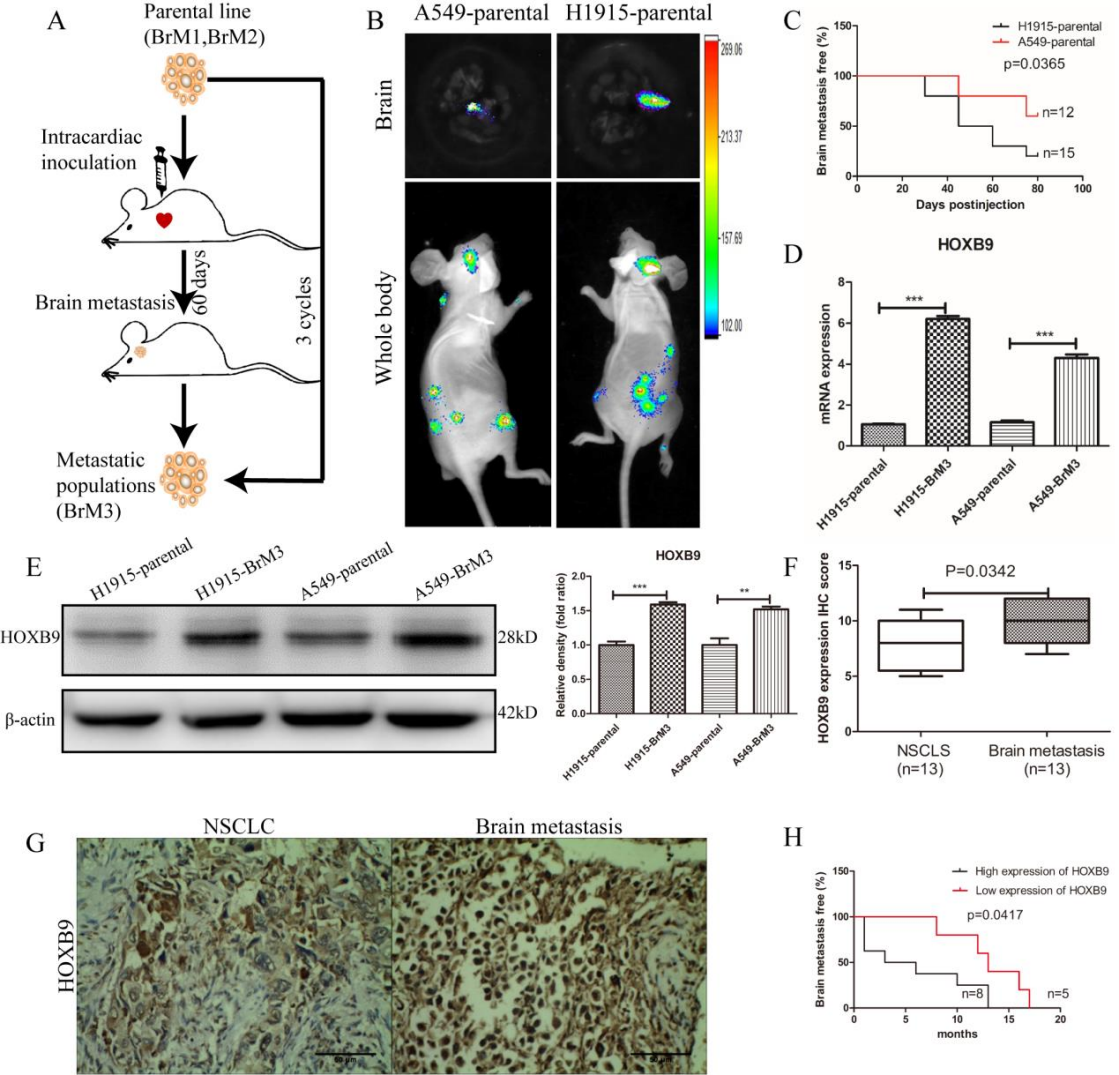
**HOXB9 silencing inhibits brain metastasis of NSCLC and prolongs brain metastasis-free survival in mice.** (**A**) *In vivo* selection scheme for the isolation of brain metastatic populations (BrM3 cells) derived from H1915 and A549 lung adenocarcinoma cell lines. (**B**) Representative bioluminescence images (whole body and brain) 60 days after intracardiac inoculation of luciferase-expressing A549 and H1915 cells. (**C**) Brain metastasis-free survival curves for mice inoculated with H1915 and A549 cells with characteristic high and low HOXB9 expression, respectively (p = 0.0365). (**D**) Comparison of relative HOXB9 mRNA expression between metastatic NSCLC cell populations (BrM3) and their parental cells. HOXB9 overexpression was confirmed in BrM3 cells (p < 0.001). (**E**) Western blotting analysis and gray level analysis of HOXB9 expression in BrM3 and parental cells. (**F**) IHC score-based quantification of HOXB9 expression in primary tumors and matched brain metastasis specimens from NSCLC patients (n = 13; p = 0.0342). (**G**) Representative images of HOXB9 expression from IHC analysis of 13 primary human NSCLC tumors and their corresponding brain metastases. Scale bars = 50 μm. (**H**) HOXB9 expression-based analysis of brain metastasis-free survival in 13 NSCLC patients that developed brain metastases. Patients with low HOXB9 expression showed longer brain metastasis-free survival (p = 0.0417). (*p < 0.05, **p < 0.01, ***p < 0.001).

Considering that HOXB9 expression increased migration and invasion potential in NSCLC cells, we hypothesized that it may facilitate brain metastasis formation. To test this hypothesis, H1915 and A549 cells, with high- and low-HOXB9-expression respectively, were transduced with the luciferase gene and injected intracardially into nude mice. The resulting brain metastases were excised, and by *in vitro* propagation and two rounds of *in vivo* selection [30] two brain metastatic NSCLC populations (A549-BrM3 and H1915-BrM3) were isolated (Figure 4A). Sixty days after tumor implantation, bioluminescence imaging (BLI) showed stronger brain metastatic activity and shorter brain metastasis-free survival in mice injected with H1915 cells, compared to those injected with A549 cells (Figure 4B, 4C). The resulting brain metastases were excised, and by *in vitro* propagation and two rounds of *in vivo* selection [30] two brain metastatic NSCLC populations (A549-BrM3 and H1915-BrM3) were isolated. Western blot and qPCR assays showed that the expression of HOXB9 in BrM3 cells was higher than in the corresponding parental cells (Figure 4D, 4E). Moreover, comparable results were obtained through IHC in matched tumor samples from primary NSCLC and brain metastases obtained from 13 clinical cases (Figure 4F, 4G and Supplementary Table 1). Among these patients, those with low HOXB9 expression (n = 5) had longer brain metastasis-free survival than those with high HOXB9 expression (n = 8) (Figure 4H). These results of animal and clinical experimentation suggest that HOXB9 has crucial role in brain metastases from non-small cell lung cancer.

### HOXB9 promotes epithelial-mesenchymal transition (EMT) in NSCLC cells and enhances their ability to cross the BBB

**Figure 5:**
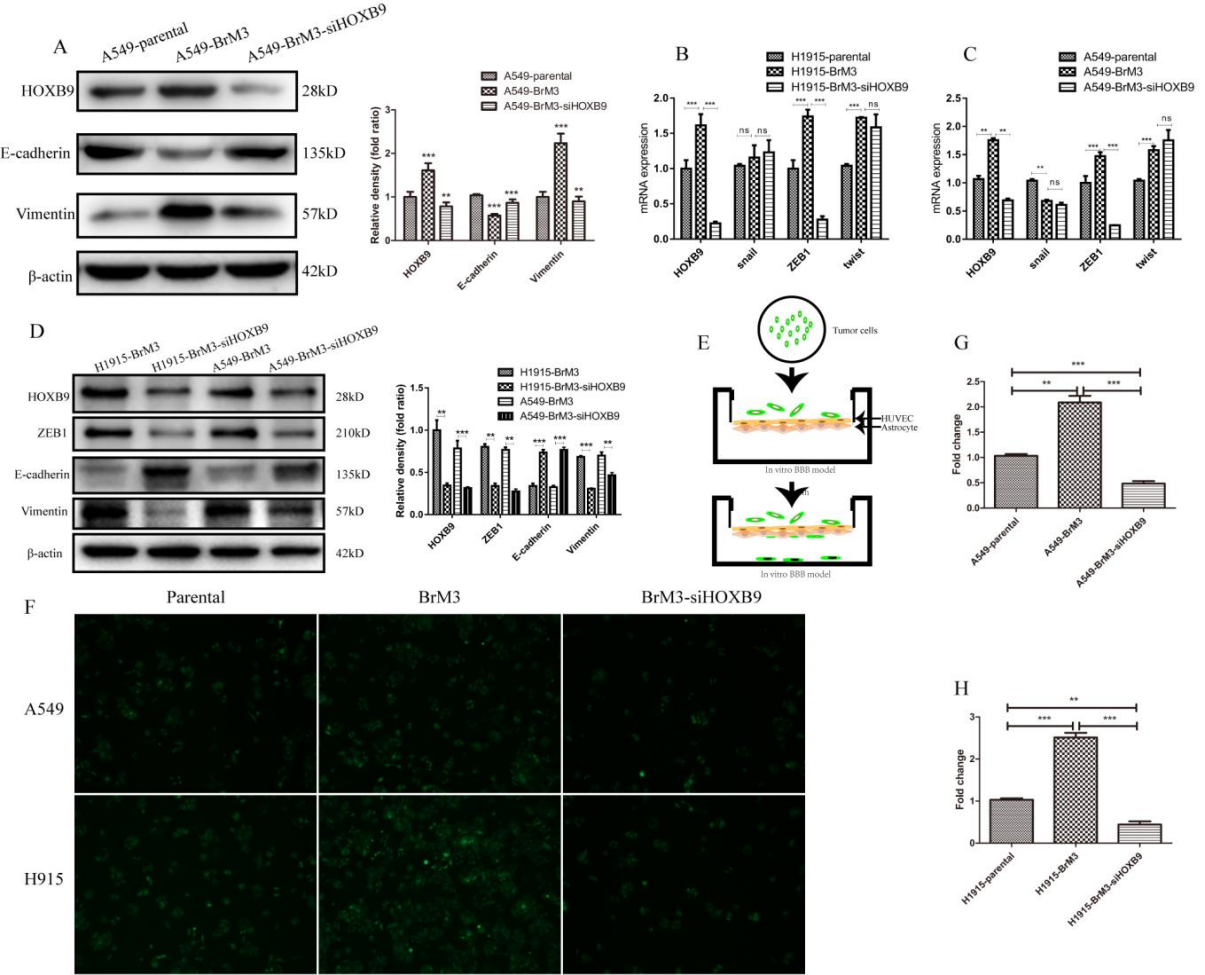
**HOXB9 knockdown inhibits EMT and weakens the ability of BrM3 cells to penetrate the BBB.** (**A**) Western blotting analysis and gray level analysis of EMT-related proteins (E-cadherin and vimentin) in BrM3 cells after HOXB9 silencing. (**B**, **C**) Relative mRNA expression of EMT-related transcription factors (snail, twist, and ZEB1) in BrM3 cells after HOXB9 silencing. (**D**) Western blotting analysis and gray level analysis of snail, twist and ZEB1 in BrM3 cells after HOXB9 silencing. (**E**) Schematic representation of the *in vitro* human BBB model. (**F**) Transmigration of NSCLC cells across the *in vitro* BBB model. Both parental cells and BrM3-siHOXB9 cells showed limited transmigratory ability. (**G**, **H**) Quantitative analysis of data obtained from the experiments shown in (**F**). *p < 0.05, **p < 0.01, ***p < 0.001.

Based on the above evidence, we speculated that HOXB9 may promote NSCLC metastasis by activating the epithelial-mesenchymal transition (EMT) program. Western blots analysis in parental A549 and H1915 cells and their metastatic counterparts (A549-BrM3 and H1915-BrM3 cells) showed that epithelial features (E-cadherin) were downregulated, while mesenchymal features (vimentin) were upregulated, in BrM3 cells. Additional analysis showed that these expression changes were reversed after HOXB9 knockdown (Figure 5A). We also assessed the expression on the EMT-driving transcription factors snail, twist, and ZEB1. Both qPCR and western blotting showed that ZEB1 was upregulated in BrM3 cells, and its expression decreased after HOXB9 silencing (Figure 5B–5D). These data indicate that HOXB9 activates EMT in NSCLC cells by inducing the expression of ZEB1.

To further verify the metastatic potential of BrM3 cells, we co-cultured human umbilical vein endothelial cells (HUVECs) and human astrocytes (HA) on opposite sides of Transwell membranes to construct an *in vitro* model of the BBB (Figure 5E). After CellTracker Green CFMDA labeling, parental A549 and H1915 cells, as well as their control- or siHOXB9-transfected BrM3 counterparts, were alternatively placed in the upper chamber (i.e onto the HUVEC monolayer) of the inserts. The lower chamber was filled with media containing 30% FBS, and after 24-h incubation the number of tumor cells crossing into the lower chamber was recorded by confocal microscopy. Compared to parental cells, the number of BrM3 cells that passed through the BBB model was significantly higher. However, this number was significantly weakened when HOXB9 was knocked down (Figure 5F–5H). According to the experimental results shown above, HOXB9 can enhance the ability of cancer cells to pass through the blood-brain barrier.

### HOXB9 disrupts BBB integrity by upregulating MMP9

**Figure 6:**
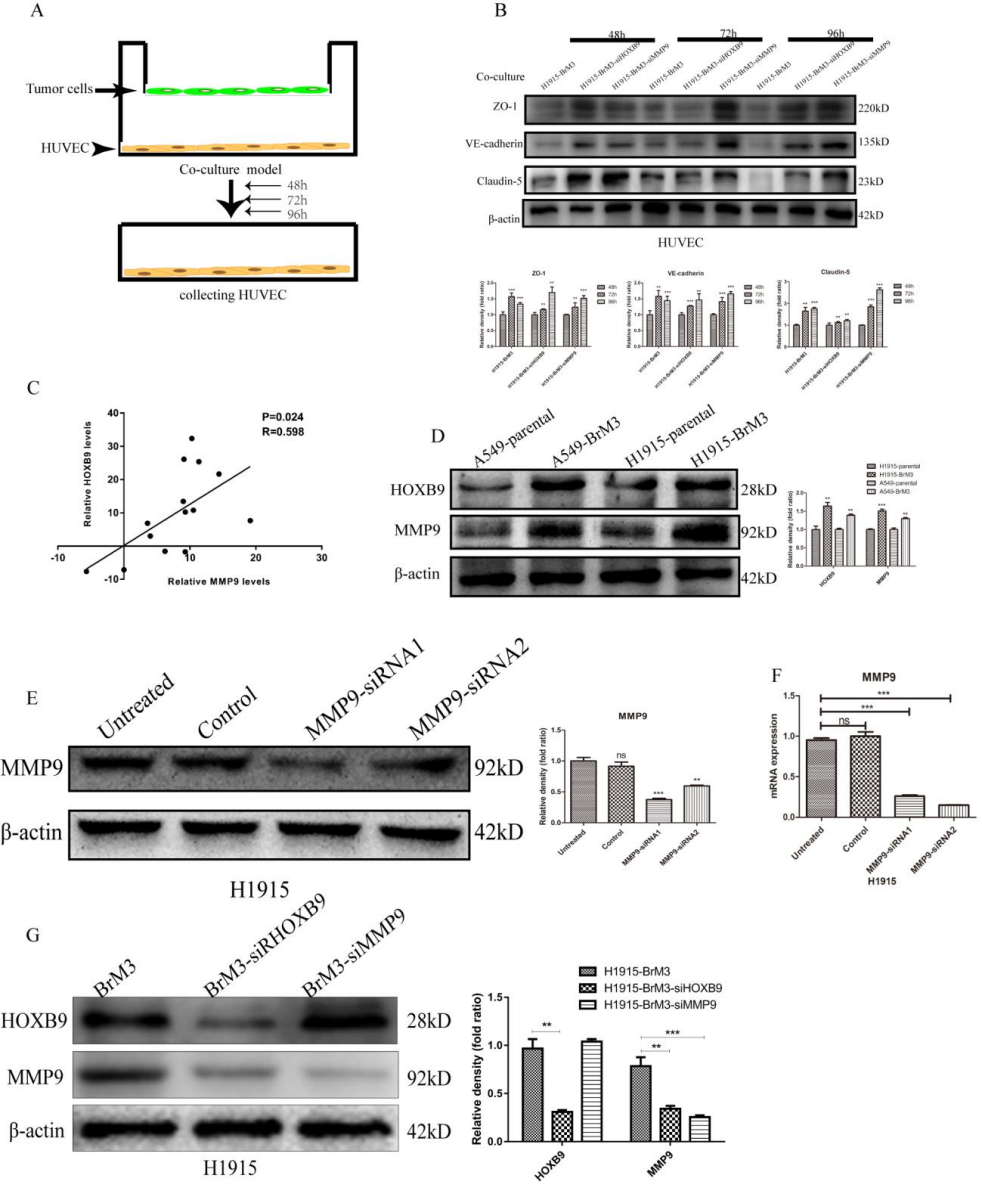
**HOXB9 mediates degradation of endothelial junctional proteins by upregulating MMP9.** (**A**) Experimental design of the HUVEC/NSCLC co-culture system. (**B**) Western blotting analysis and gray level analysis showing restored expression of junctional proteins (ZO-1, claudin-5 and VE-cadherin) in HUVECs co-cultured with BrM3 cells in which HOXB9 or MMP9 were silenced. (**C**) Analysis of MMP9 and HOXB9 co-expression in primary NSCLC and matched brain metastases from the GSE74706 dataset (p = 0.024, r = 0.598). (**D**) Western blotting analysis and gray level analysis of MMP9 and HOXB9 expression in parental NSCLC cell lines and corresponding BrM3 cells. (**E**, **F**) Western blot analysis, gray level analysis and qPCR assays of MMP9 expression in H1915 cells transfected with MMP9-siRNA or control siRNA. (**G**) Western blotting analysis and gray level analysis of MMP9 and HOXB9 expression in H1915 cells in which HOXB9 or MMP9 were silenced. *p < 0.05, **p < 0.01, ***p < 0.001.

Junctional proteins play a critical role in the regulation of BBB permeability and their downregulation or destruction are associated with disruption of BBB integrity. To evaluate the impact of HOXB9 expression in tumor cells on junctional proteins involved in endothelial barrier function, we examined the expression of ZO-1, claudin-5, and VE-cadherin in HUVECs after co-culture with tumor cells (Figure 6A). The results showed that the expression of the three proteins was significantly downregulated after co-culture with BrM3 cells (showing high HOXB9 expression). In contrast after co-culture with BrM3-siHOXB9 cells, ZO-1, claudin-5 and VE-cadherin expression increased significantly (Figure 6B). These results indicate that high expression of HOXB9 in tumor cells may compromise BBB integrity by leading to disruption of adhesion junctions between adjoining endothelial cells.

Matrix metalloproteinase 9 (MMP9), a member of the zinc- and calcium-dependent MMP family with endopeptidase activity, can degrade most extracellular matrix components and decrease the expression of junctional proteins to increase BBB permeability. Therefore, we examined through western blot whether MMP9 expression is upregulated by HOXB9 overexpression in NSCLC cells. As shown in Figure 6D, higher MMP9 expression was detected in BrM3 cells (showing high HOXB9 expression) compared with their parental counterparts. Moreover, this result was consistent with analysis of MMP9 and HOXB9 co-expression in primary NSCLC from a public GEO dataset (GSE74706; Figure 6C). To verify these data, HUVECs were co-cultured with BrM3 cells in which MMP9 was knocked down via siRNA (Figure 6E, 6F). Western blot assays showed that MMP9 silencing in BrM3 cells restored the expression of ZO-1, claudin-5, and VE-cadherin in HUVECs (Figure 6B). We found that the expression of MMP9 was downregulated by HOXB9 silencing, but MMP9 silencing had little effect on the expression of HOXB9 (Figure 6G). These data suggest that HOXB9 is a positive regulator of MMP9 expression in NSCLC cells, and underscore a potential mechanism by which HOXB9 overexpression promotes metastatic spread of NSCLC cells to the brain.

## DISCUSSION

Homeobox B9 (HOXB9) belongs to a highly conserved family of HOX transcription factor genes that are essential for embryonic development. In cancer, the expression of the hox gene complex directly drives tumor transformation and progression by inhibiting apoptosis, altering receptor signaling, and promoting EMT and invasion [32]. Several studies highlighted a role for HOXB9 in cancer progression, although both pro-tumoral and anti-tumoral effects have been described in different cancer types. For example, high HOXB9 expression was shown to promote EMT via TGF-β signaling and was associated with shorter survival in hepatocellular [33] and oral squamous cell [34] carcinomas. In contrast, HOXB9 was reported to induce tumor cell differentiation via mesenchymal-to-epithelial transition (MET) and was correlated with favorable prognosis in gastric carcinoma [35]. Intriguingly, elevated HOXB9 expression has been linked to both favorable and poor prognosis in colon cancer patients [23] [24, 36]. Therefore, the function of HOXB9 deserves further scrutiny as it appears to be dependent on cancer type and/or stage.

Lung cancer is well established as a highly heterogeneous tumor that harbors various gene alterations including mutations in EGFR, KRAS, BRAF, PD-L1, and PIK3CA, as well as ALK rearrangement and HER2 amplification. We analyzed these abnormal genes and HOXB9 mRNA expression in NSCLC and normal lung tissues from GEO public databases (GSE74706) and found that the expression levels of HOXB9, EGFR, KRAS and BRAF were significantly increased in NSCLC (Supplementary Figure 2A). This finding suggested that HOXB9 functions as an oncogene in NSCLC. Correlation analyses revealed a significant positive correlation between the expression levels of ALK and HOXB9 (Supplementary Figure 2B). Patients with ALK-positive NSCLC are more likely to present HOXB9 overexpression. However, HOXB9 is negatively correlated with PD-L1 expression (Supplementary Figure 2C), so it is speculated that the use of PD-L1 inhibitors in NSCLC with high HOXB9 expression may not benefit significantly. But this speculation needs to be confirmed by further experiments.

Previous studies have shown that high expression of HOXB9 in NSCLC predicts poor prognosis and promotes EMT and bone and brain metastasis [29, 30]. In this study, we confirmed that HOXB9 expression is elevated in clinical NSCLC samples compared with matched normal ones. In addition, high HOXB9 expression promotes migration and invasion of NSCLC cells without affecting their proliferative potential. Therefore, HOXB9 is an important oncogene in the development of NSCLC, and it can be used as an indicator of poor prognosis for patients.

Moreover, the metastatic potential imparted by HOXB9 overexpression was corroborated *in vivo*. In addition, IHC analysis showed higher HOXB9 expression in patient-derived NSCLC brain metastases compared to matched primary tumor specimens. This illustrates that HOXB9 enhances the ability of lung cancer to develop brain metastases. Further evidence linking HOXB9 expression and metastatic capacity came from demonstration that cell populations derived from brain metastasis-forming H1915 and A549 cells after two rounds of *in vivo* selection (BrM3 cells) showed markedly higher levels of HOXB9 than their parental counterparts. These results are consistent with a previous study that showed that WNT/TCF signaling through LEF1 and HOXB9 mediates lung adenocarcinoma metastasis to brain and bone [30], however, the specific mechanisms had not been fully elucidated so far.

EMT is a strictly controlled process that is critical for a variety of physiological and pathological events such as embryonic morphogenesis, fibrotic disease, and tumor metastasis [37]. EMT enhances cancer cells’ motility and ability to escape apoptosis, favoring dissemination and formation of metastatic tumors [38, 39]. We found that HOXB9 can enhance the EMT process of NSCLC, which makes it easier for tumor cells to spread into the blood circulation, and eventually colonize the tumor to distant organs. This may be one of the reasons why HOXB9 promotes brain metastasis of NSCLC.

Taken together, this study provide a better understanding of the oncogenic mechanism of HOXB9, which can induce EMT of tumor cells and destroy the integrity of the BBB. Brain metastasis occurs after tumor cells spreading from a primary tumor reach the brain microvasculature through the blood [40]. The brain is protected by the BBB, which limits the penetration of macromolecules and cells [40]. Specialized protein complexes composed of tight junction proteins such as ZO-1, JAMs, occludin, and claudins (1,3,5,12), and adhesion junction proteins including VE-cadherin, PECAM-1 and nectin, mediate intercellular adhesion between contiguous vascular endothelial cells and determine the highly selective permeability characteristic of the BBB [41, 42]. Our data showed that HOXB9 expression positively regulated the ability of NSCLC cells to migrate through an *in vitro* BBB model composed of vascular endothelial cells (HUVECs), and astrocytes (HA) of human origin [12, 43]. HOXB9 overexpressed in tumor cells can destroy the integrity of the BBB by degrading junctional proteins (ZO-1, claudin-5, and VE-cadherin), and promote tumor cells to cross the BBB. In turn, knockdown assays in NSCLC cells using siRNAs indicated that this effect was secondary to enhanced MMP9 expression, which correlated with reduced expression of junctional proteins in HUVECs. MMP9 is a main endopeptidase mediating BBB disruption in various lesional and pathological processes, including ischemic stroke [44], and viral infection [45]. Since the expression of junctional proteins in HUVECs was restored after MMP9 knockdown in Br3M cells, we propose that HOXB9 drives MMP9 expression to destroy the integrity of the BBB and facilitate metastatic spreading of NSCLC cells.

**Figure 7:**
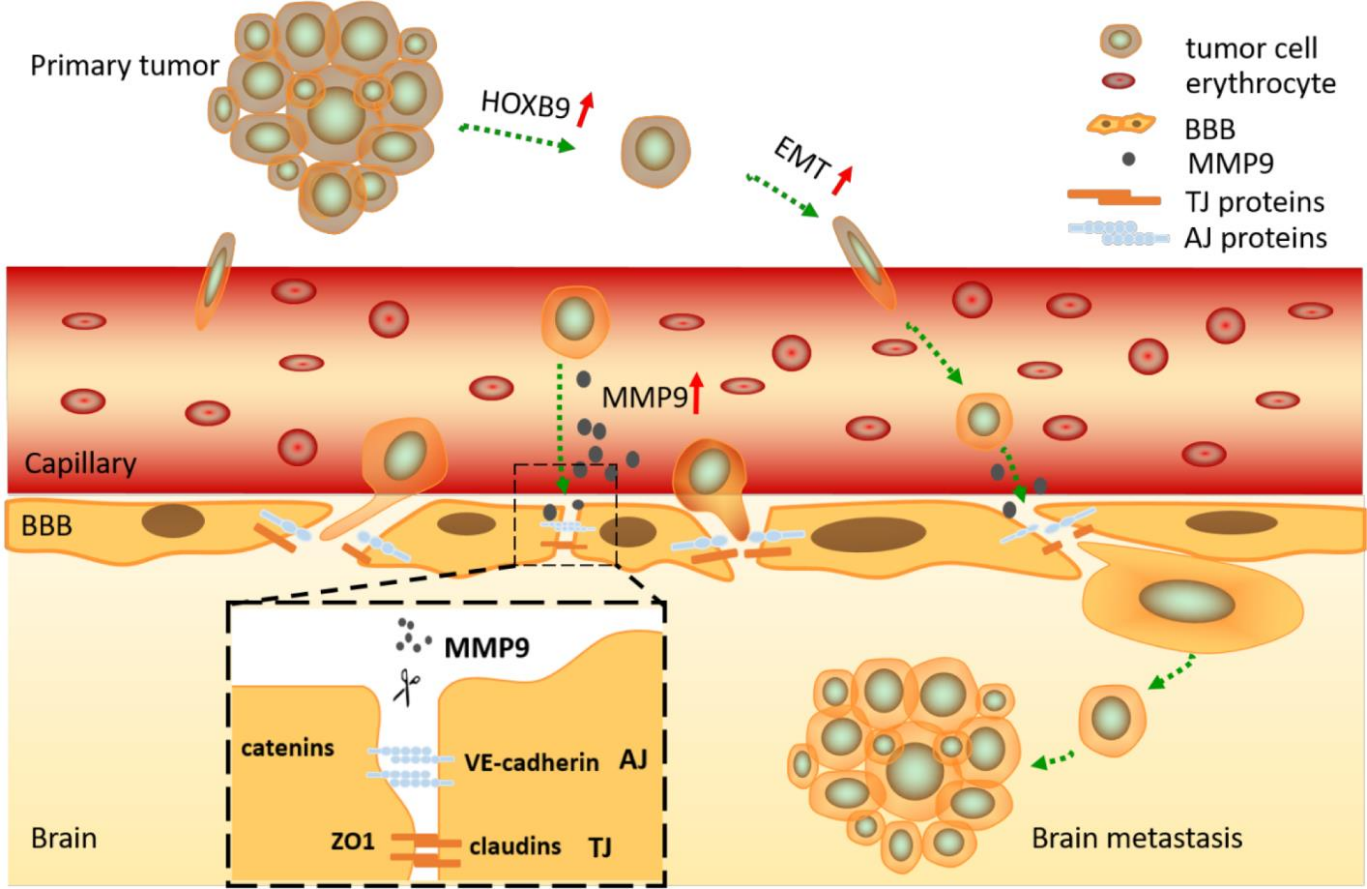
**Graphical summary.** HOXB9 promotes migration and invasion of NSCLC cells by inducing EMT, allowing them to break away from the primary bulk tumor and invade the surrounding capillaries. After reaching the cerebral circulation, the cells are arrested at the BBB, where degradation of junctional proteins (TJ and AJ) occurs due to release of MMP9 by tumor cells in a HOXB9-dependent manner. This results in BBB breakdown, tumor cell invasion, and establishment of brain metastasis.

In summary, our research suggests that HOXB9 expression in NSCLC cells promotes BBB disruption and mediates invasion into the brain parenchyma by inducing EMT and upregulating MMP9 expression (Figure 7). While future work is needed to determine if a similar mechanism operates to facilitate metastatic dissemination of primary NSCLC tumors to other organs, and to verify the correlation between HOXB9 and ALK and PD-L1 in NSCLC. And our data suggest that HOXB9 represents a promising therapeutic target for preventing brain metastasis from primary NSCLC.

## MATERIALS AND METHODS

### Cell lines and tissue samples

Human NSCLC cell lines (PC9, A549, H1915, H1975, H1650, and H1299) and human umbilical vein endothelial cells (HUVECs) were purchased from the American Type Culture Collection (Manassas, VA, USA). Human bronchial epithelial cells (HBE) and human astrocytes (HA) were purchased from the Cell Bank of Academia Sinica (Shanghai, China). A549, A549-BrM3, H1915, H1915-BrM3, H1975, H1650, H1299, HUVEC and HBE cells were cultured in RPMI-1640, and PC9 and HA cells were maintained in DMEM (HyClone, Logan, UT, USA). These media were supplemented with 10% fetal bovine serum (Gemini, Bridgewater Township, NJ, USA), 100 μg/mL streptomycin, and 100 U/mL penicillin.

Matched samples of NSCLC, normal lung tissue, and brain metastases were obtained from surgical patients at the Harbin Medical University Cancer Hospital. Detailed explanation of the research program was given to patients and informed consent was signed. Both NSCLC and brain metastasis samples were confirmed histologically. See Supplementary Table 1. for details. The GEO GSE74706 dataset was used for data validation through correlation analysis. We used the UALCAN online tool (http://ualcan.path.uab.edu/ind ex.html) to analyze the expression of HOXB9 in NSCLC in TCGA database. The present study was approved by the Ethics Committee of Harbin Medical University Cancer Hospital.

### Gene knockdown and overexpression

HOXB9 and MMP9 were silenced via specific siRNAs, using scrambled siRNAs as control. A vector encoding Flag-HOXB9 was used to induce HOXB9 overexpression in A549 cells, and an empty vector served as a control. Transfection was carried out using Lipofectamine reagents (Life Technologies, Carlsbad, CA, USA) according to the manufacturer’s instructions. The details of qPCR primers and siRNA sequences are in Supplementary Table 2.

### Animal experiments

Animals (BALB/c athymic nude mice, male, 4–6 weeks old) were bought from Beijing Vital River Laboratory. Tumor cells (A549 and H1915) were labelled with luciferase-expressing lentiviral particles. To construct the brain metastasis model, 10^5^ tumor cells suspended in 100 μl PBS were inoculated into the left cardiac ventricle. To isolate brain metastatic cell populations (i.e. A549-BrM3 and H1915-BrM3) two rounds of *in vivo* selection were performed after initial isolation and *in vitro* culturing of A549 and H1915 cells forming brain metastases [30]. *In vivo* bioluminescence was used to detect metastasis formation and to construct brain metastasis-free survival curves (reflecting the time elapsed from tumor cell injection until detection of brain metastasis). All animal experiments were performed in accordance with the Institutional Animal Care and Use Committee (IACUC) of Harbin Medical University in China and the NIH Guide for the Care and Use of Laboratory Animals.

### *In vitro* BBB model and transcellular migration assay

We used HUVEC and HA cells plated on Transwell inserts to construct an *in vitro* blood-brain barrier model [12, 43]. Twenty-four-well Transwell polycarbonate inserts with 3-μm pore size (BD Biosciences, San Jose, CA, USA) were coated with 2% gelatin for 45 min. The inserts were placed upside down and 10^5^ astrocytes were seeded at the bottom of the insert. Cells were incubated (37 ° C, 5% CO_2_) for 3 hours and fed every 15-30 min. The inserts were then placed in a 24-well plate containing 1 ml of media, and astrocyte growth was induced by further culturing for 1 day. Then, 100,000 HUVECs were seeded in the insert’s upper chamber surface, and the culture was maintained for another 3 days. The permeability of the modeled BBB was verified by adding Trypan blue to the upper chamber and measuring after a 30-min incubation at 37° C the absorbance (595 nm) of extravasated dye. Duplicate cultures were tested in each experiment.

To study the ability of tumor cells to cross the BBB model described above, NSCLC cells labeled with 5 μM of CellTracker Green CFMDA (Invitrogen, Carlsbad, CA, USA) in serum-free medium for 45 min were inoculated (10,000 cells in 200 μl of DMEM medium containing 3% FBS) into the upper chamber of the inserts. The lower chamber was filled with 500 μl of DMEM medium containing 30% FBS. After overnight incubation, the inserts were removed and the number of cells that crossed to the lower chamber was recorded by confocal microscopy.

### Quantitative RT-PCR analysis

Total RNA from tumor cells was extracted using TRIzol reagent (Invitrogen). The RNA template was reverse-transcribed into cDNA using Transcriptor First Strand cDNA Synthesis Kit (TransGen, Beijing, China). Quantitative analysis of HOXB9 expression was conducted using Top Green qPCR SuperMix (TransGen). All samples were tested three times and target gene expression changes relative to GAPDH were calculated based on threshold cycle (Ct) analysis. The details of qPCR primers and siRNA sequences are in Supplementary Table 2.

### Western blot and immunohistochemistry

Western blot and IHC were performed as described by us previously [46]. The following antibodies were used: anti-HOXB9, anti-ZO1, anti-VE-cadherin, anti-Claudin5 and anti-MMP9 (Abcam, Cambridge, MA, USA), anti-ZEB1, anti-E-cadherin, anti-vimentin, anti-Flag, and anti-β-actin (Proteintech, Wuhan, Hubei, P.R.C). See Supplementary Table 3. for details. The original data of all WB protein expression is attached in the Supplementary Figures 3–5.

### Statistical analysis

SPSS 18.0 and GraphPad Prism software were used in statistical analysis. Each experiment was performed at least three times. Data were analyzed by one-way ANOVA or Student’s t test and are expressed as mean ± standard deviation (SD) or standard error of the mean (SEM). P < 0.05 was considered significant.

## Supplementary Material

Supplementary Figures

Supplementary Tables
