# Supplementary material for: HOXB9 enhances the ability of lung cancer cells to penetrate the blood-brain barrier
Source: Aging (Albany NY). 2020 Dec 19;13(4):4999–5019. doi: 10.18632/aging.202324 (PMC7950248; doi:10.18632/aging.202324)
Supplement: Supplementary Figures [file aging-13-202324-s001.pdf]

SUPPLEMENTARY FIGURES

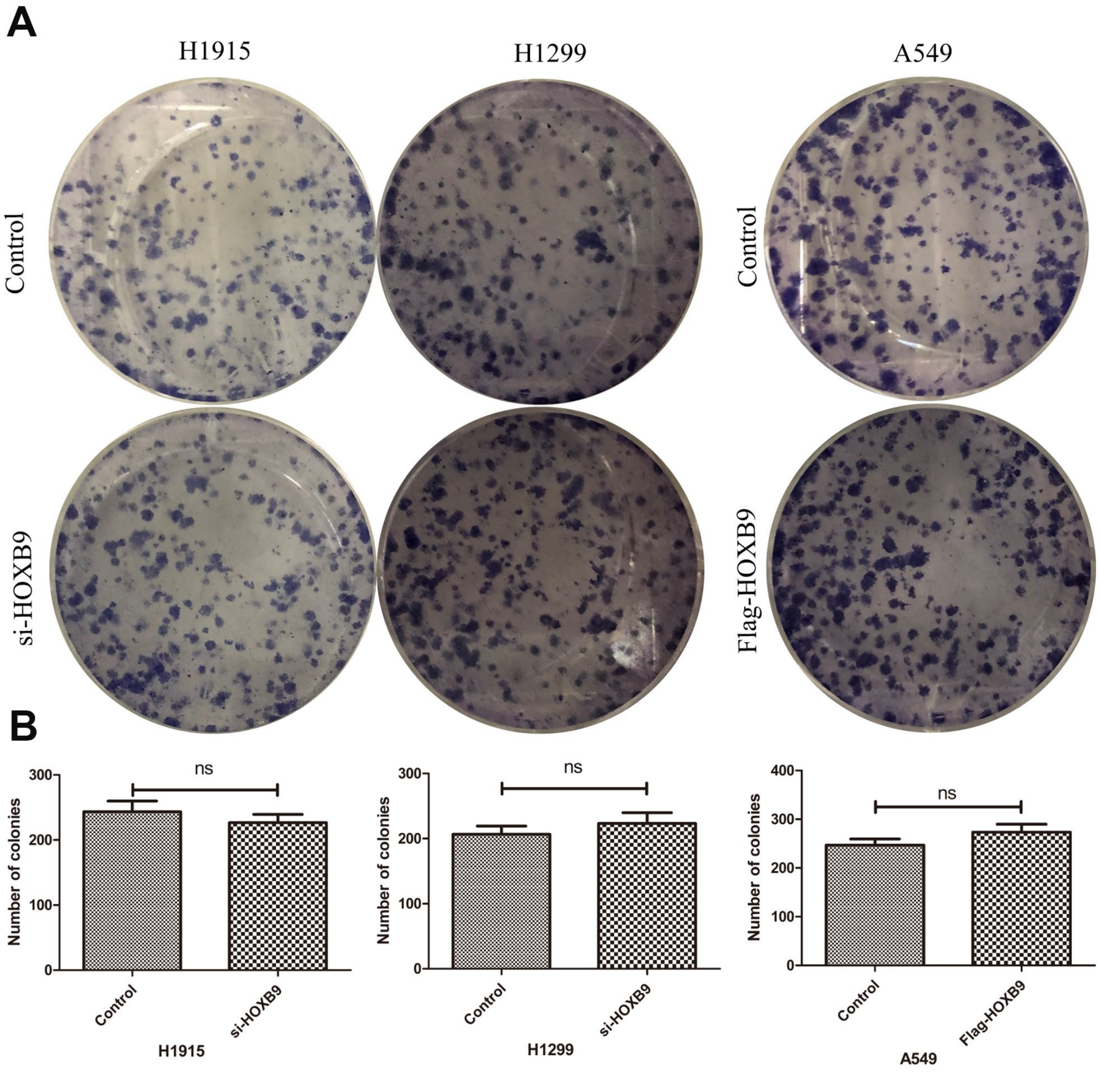

Supplementary Figure 1. HOXB9 expression does not affect colony formation efficiency in NSCLC cells. (**A**) Colony forming efficiency (CFE) assays were conducted to assess the effect of HOXB9 expression on the proliferation of NSCLCs. (**B**) Quantification of CFE assay data.

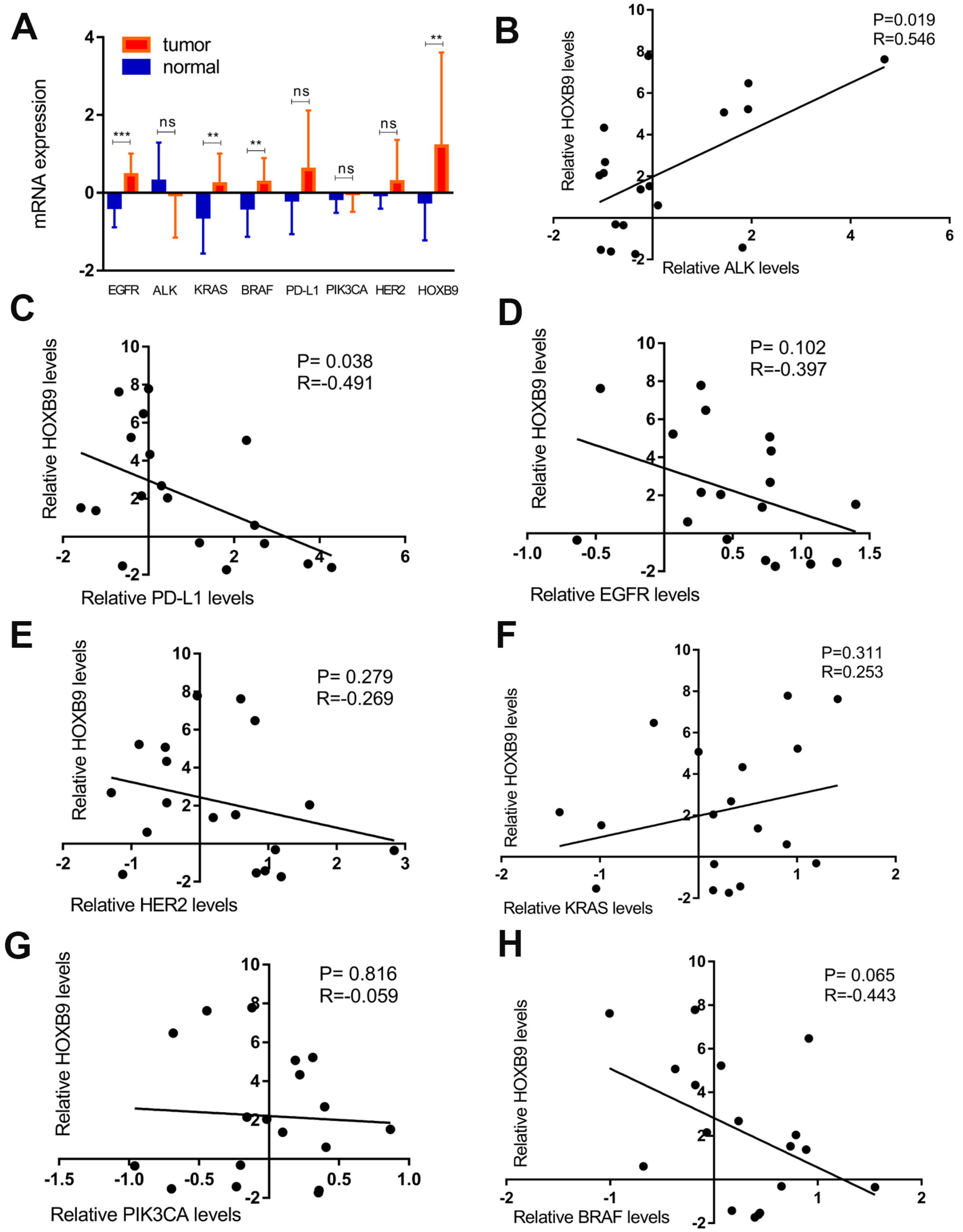

Supplementary Figure 2. The expression relationship between HOXB9 and common abnormal genes in lung cancer through GEO database (GSE74706). (**A**) The expression levels of HOXB9, EGFR, KRAS and BRAF were significantly increased in NSCLC. (**B**) Correlation analyses revealed a significant positive correlation between the expression levels of ALK and HOXB9. (**C**) HOXB9 is negatively correlated with PD-L1 expression. (**D**–**H**) These pictures show the correlation between HOXB9 and EGFR, HER2, KRAS, PIK3CA,BRAF, but not statistically significant. *p < 0.05, **p < 0.01, ***p < 0.001.

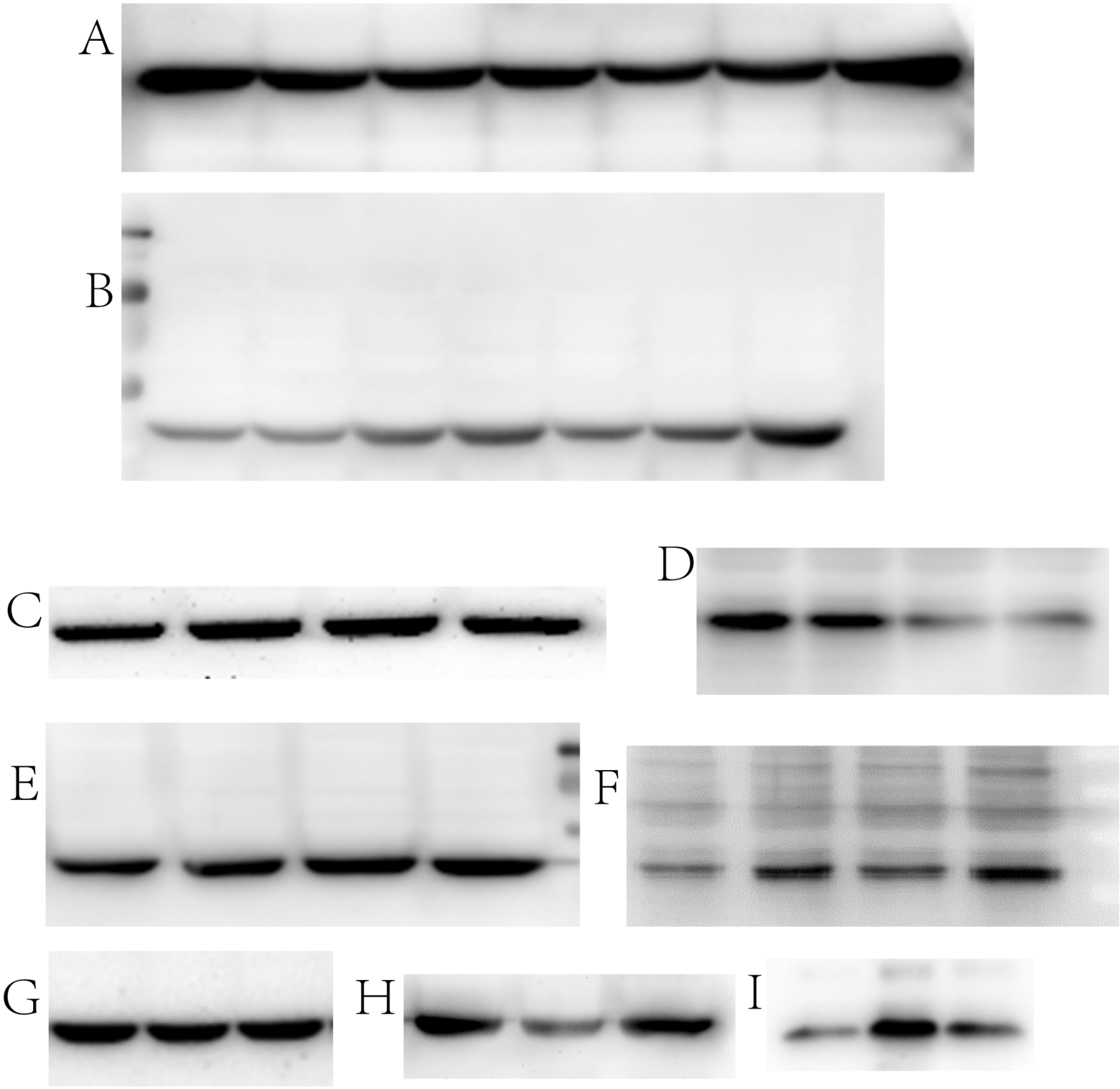

Supplementary Figure 3. The original data of WB protein expression. (**A**, **B**) The original data of WB protein expression of Figure 1F. (**C**, **D**) The original data of WB protein expression of Figure 2A. (**E**, **F**) The original data of WB protein expression of Figure 4E. (**G**–**I**) The original data of WB protein expression of Figure 5A.

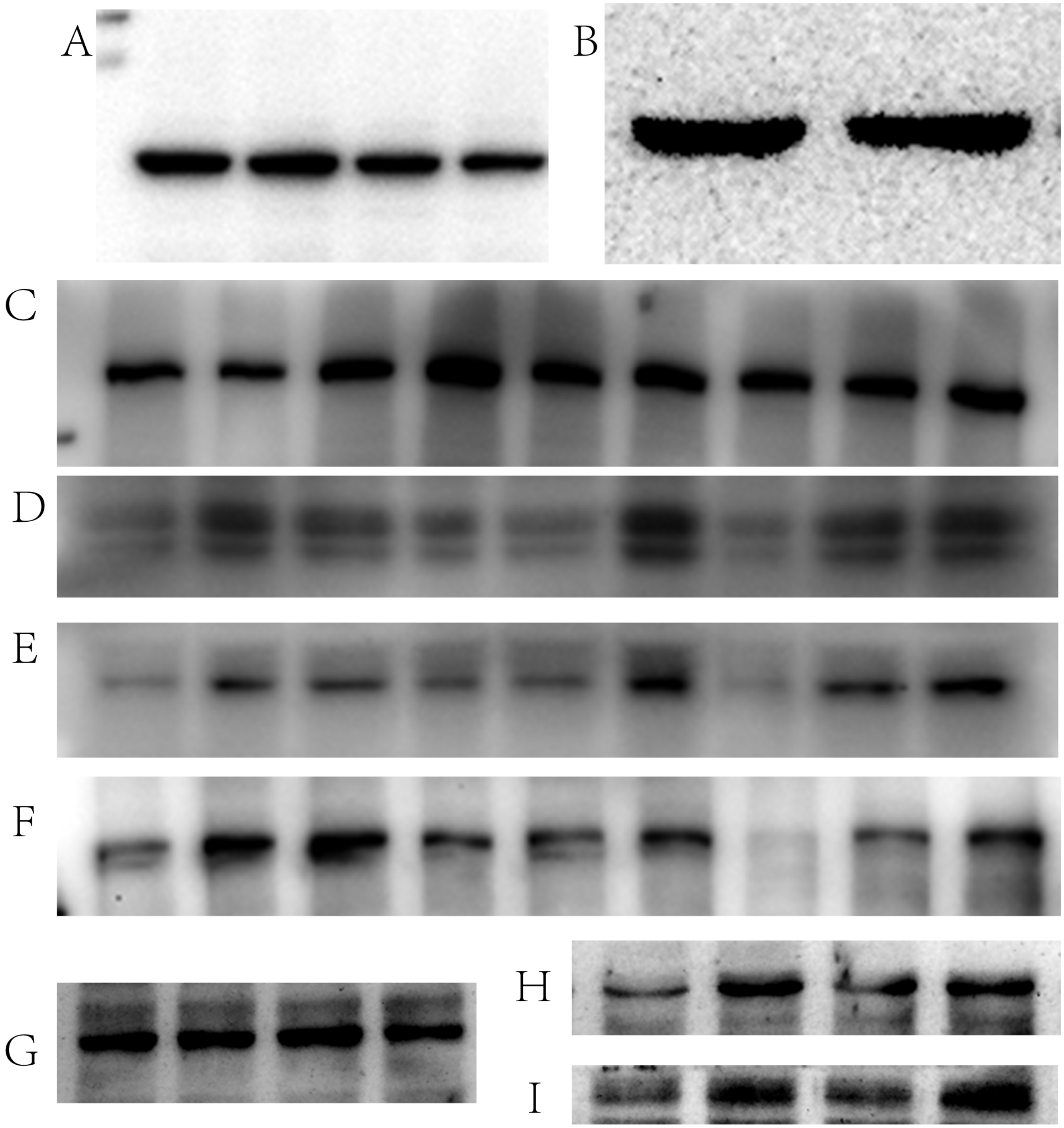

Supplementary Figure 4. The original data of WB protein expression. (**A**) The original data of WB protein expression of Figure 5D. (**B**) The original data of WB protein expression of Figure 3A. (**C**–**F**) The original data of WB protein expression of Figure 6B. (**G**–**I**) The original data of WB protein expression of Figure 6D.

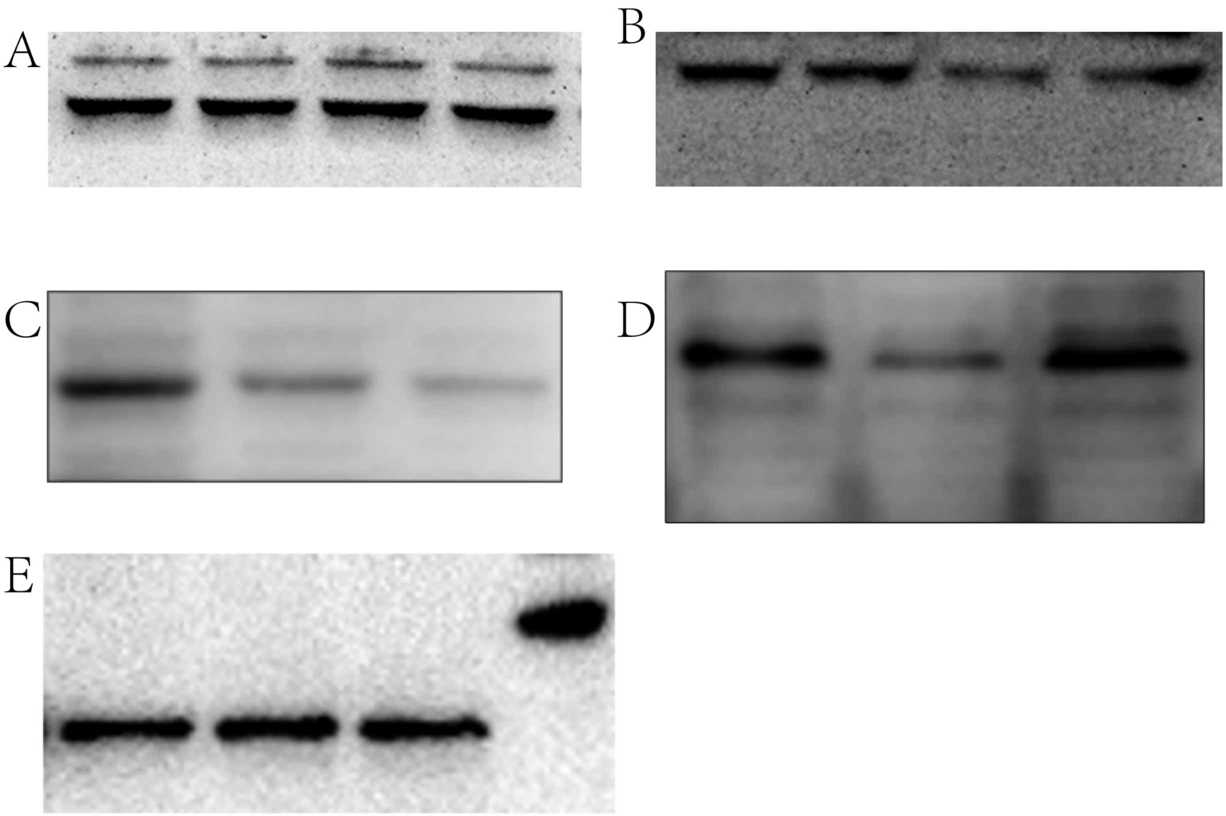

Supplementary Figure 5. The original data of WB protein expression. (**A**, **B**) The original data of WB protein expression of Figure 6E. (**C**–**E**) The original data of WB protein expression of Figure 6G.
