# Supplementary material for: HOXB9 enhances the ability of lung cancer cells to penetrate the blood-brain barrier
Source: Aging (Albany NY). 2020 Dec 19;13(4):4999–5019. doi: 10.18632/aging.202324 (PMC7950248; doi:10.18632/aging.202324)
Supplement: Supplementary Tables [file aging-13-202324-s002.pdf]

SUPPLEMENTARY TABLES

Supplementary Table 1. Clinicopathological features of patients (N=13) with brain metastases of NSCLC.

Characteristic	No. of cases	%
**HoxB9 expression**		
**Low**	5	38.5
**High**	8	61.5
**Sex**		
**Male**	7	53.8
**Female**	6	46.2
**Age (years)**		
**<60**	8	61.5
**≥60**	5	38.5
**Pathological type**		
**Adenocarcinoma**	10	76.9
**Squamous cell carcinoma**	3	23.1
**Brain metastasis-free survival (months)**		
**≤12**	9	69.2
**>12**	4	30.8

Supplementary Table 2. qPCR primers and siRNA sequences.

Gene symbol	Sequences
HOXB9	forward:5'-CCATTTCTGGGACGCTTAGCA-3',<br>reverse: 5'-TGTAAGGGTGGTAGACGGACG-3'.
MMP9	forward:5'-GCGCTGGGCTTAGATCATTC -3',<br>reverse: 5'- AGGTGCCGGATGCCATT-3'.
ZEB1	forward:5'- AAGTGGCGGTAGATGGTAATGT -3',<br>reverse: 5'- AAGGAAGACTGATGGCTGAAAT-3'.
SNAIL	forward:5'- CTCGGACCTTCTCCCGAATG -3',<br>reverse: 5'- AAAGTCCTGTGGGGCTGATG -3'.
TWIST	forward:5'-GCCAGGTACATCGACTTCCTCT -3',<br>reverse: 5'-TCCATCCTCCAGACCGAGAAGG -3'.
GAPDH	forward: 5'-CAATGACCCCTTCATTGACC-3',<br>reverse: 5'-GACAAGCTTCCCGTTCTCAG-3'.
Si-HOXB9	siRNA1: 5'-CCCTTCAATTTGTAGACTCTT-3',<br>siRNA2: 5'-CTCCTCAATCTGAGTGAGAGA-3'.
Si-MMP9	siRNA1: 5'-GTACCGCTATGGTTACACT-3',<br>siRNA2: 5'-GCAACGTGAACATCTTCGA-3'.

Supplementary Table 3. Primary antibodies used for western blot analysis.

Antibodies	Species	Manufacture	Catalog#	Dilution
HOXB9	Rabbit	Abcam	ab133701	1:1000
Flag	Mouse	Proteintech	60002-1-Ig	1:1000
E-cadherin	Rabbit	Proteintech	20874-1-AP	1:500
Vimentin	Rabbit	Proteintech	20874-1-AP	1:1000
ZEB1	Rabbit	Proteintech	22018-1-AP	1:1000
ZO-1	Mouse	Abcam	Ab61357	1:1000
VE-cadherin	Rabbit	Abcam	ab205336	1:1000
Claudin-5	Rabbit	Abcam	ab131259	1:1000
MMP9	Mouse	Abcam	Ab58803	1:1000
β-actin	Mouse	Proteintech	60008-1-lg	1:1000
